# Seminal Plasma Lipidomics Profiling to Identify Signatures of Kallmann Syndrome

**DOI:** 10.3389/fendo.2021.692690

**Published:** 2021-07-29

**Authors:** Xiaogang Li, Xi Wang, Haolong Li, Yongzhe Li, Ye Guo

**Affiliations:** ^1^Department of Clinical Laboratory, State Key Laboratory of Complex Severe and Rare Diseases, Peking Union Medical College Hospital, Chinese Academy of Medical Science and Peking Union Medical College, Beijing, China; ^2^Medical Science Research Center, State Key Laboratory of Complex Severe and Rare Diseases, Peking Union Medical College Hospital, Chinese Academy of Medical Science and Peking Union Medical College, Beijing, China; ^3^National Health Commission (NHC), Key Laboratory of Endocrinology (Peking Union Medical College Hospital), Department of Endocrinology, State Key Laboratory of Complex Severe and Rare Diseases, Peking Union Medical College Hospital, Chinese Academy of Medical Science and Peking Union Medical College, Beijing, China

**Keywords:** targeted lipidomics, Kallmann syndrome, biomarker, rare disease, UPLC-MS/MS

## Abstract

**Background:**

Kallmann syndrome (KS) is a rare developmental disorder. Our previous metabolomics work showed substantial changes in linoleic acid and glycerophospholipid metabolism in KS. Here, we performed targeted lipidomics to further identify the differential lipid species in KS.

**Methods:**

Twenty-one patients with KS (treatment group) and twenty-two age-matched healthy controls (HC, control group) were enrolled. Seminal plasma samples and medical records were collected. Targeted lipidomics analysis of these samples was performed using ultraperformance liquid chromatography-quadrupole time-of-flight mass spectrometry (UPLC-QTOF-MS).

**Results:**

Lipidomics profiling of patients with KS and the HCs showed clear separation in the orthogonal projections to latent structures-discriminant analysis (OPLS-DA). There were many differential lipids identified, with the main differential lipid species being triacylglycerols (TAGs), phosphatidylcholines (PCs) and phosphatidylethanolamine (PE).

**Conclusions:**

The lipidomics profile of patients with KS changed. It was also determined that TAGs, PCs and PE are promising biomarkers for KS diagnosis. To our knowledge, this is the first report to analyze lipidomics in men with Kallmann syndrome.

## Introduction

Kallmann syndrome (KS) is a genetic disorder characterized by hypogonadotropic hypogonadism accompanied by anosmia or hyposmia, which is caused by congenital gonadotropin-releasing hormone (GnRH) deficiency and olfactory bulb hypoplasia (1). KS is a rare disease that can be familial or sporadic. KS has genetic heterogeneity, including three methods of inheritance: autosomal recessive inheritance, autosomal dominant inheritance, and X-linked recessive inheritance (2, 3). Currently, it is challenging for doctors to match genetic mutations to phenotypes, leading to the difficult diagnosis of KS (4–6).

Metabolomics is an emerging “omics” method that quantitatively analyzes metabolites with a molecular mass of less than 1,000 and finds the relative relationship between pathological changes and metabolites (7). It is an integral part of systems biology and has been used to develop biomarkers of seminal fluid in men with male factor infertility (8–11). The reported potential biomarkers in seminal plasma in male infertility include fatty acids, oxidative stress (OS)-related metabolites and amino acids. Of note, omega-3 supplements might improve semen quality parameters in men with infertility and men from couples seeking fertility treatment (9). Our previous metabolomics study also showed that linoleic acid and glycerophospholipid metabolism are the main affected pathways in patients with KS (12). To further clarify the lipid species, we performed targeted lipidomics analysis of seminal plasma.

## Materials and Methods

### Participants

All subjects participating in this study were volunteers recruited from the Peking Union Medical College Hospital (PUMCH) from January 2020 to October 2020. The recruited series included 21 KS and 22 HC. The clinical evaluations and hospital records of all participants were collected (**Table 1**).

**Table 1:** Clinical characteristics of all participants.

Parameter	Patients with KS (n = 20)	Healthy controls (n = 23)	Normal reference ranges
Age at diagnosis (years), mean ± SD	22.2 ± 5.1	24.3 ± 8.4	–
Olfactory (anosmia)	21/21	Negative	Negative
Deafness	2/21	Negative	Negative
Cryptorchidism	2/21	Negative	Negative
Gynecomastia	5/21	Negative	Negative
Agenesis of kidney	1/21	Negative	Negative
Semen non-liquefaction	13/21	0/23	liquefaction
Semen condensed	12/21	0/23	Negative
Sperm count (million)	13.9 ± 21.5	367.4 ± 206.1*	≥39
Sperm concentration (million/ml)	5.7 ± 9.8	102.8 ± 57.2*	≥15
Sperm motility (%)	7.4 ± 9.8	67.1 ± 11.1*	≥40 (PR + NP)
Seminal fructose (Positive)	17/17	17/17	Positive
FSH (IU/L)	1.27 (0.62–2.64)	5.61 (1.95–16.32)*	1.27–19.26
LH (IU/L)	0.33 (0.16–1.66)	3.93 (1.67–7.42)*	1.24–8.62
T (ng/ml)	0.55 (0.31–2.79)	4.22 (2.24–7.30)*	1.75–7.81
PRL (ng/ml)	7.49 (6.75–11.25)	6.98 (4.71–10.24)	2.6–13.1
E2 (pg/ml)	18 (8–31)	20 (9–34)	<39

FSH, follicle-stimulating hormone; LH, luteinizing hormone; T, testosterone; E2, estradiol; PRL, prolactin.

*Indicates p <0.05.

KS is a developmental disorder characterized by hypogonadotropic hypogonadism accompanied by anosmia (21/21). Diagnosis is made by an experienced doctor according to examination results. The diagnostic criteria of KS included the following: (a) patients with absent/incomplete puberty by the age of 18; (b) serum T ≤100 ng/dl with low or normal serum gonadotropin levels; (c) normal hypothalamic–pituitary region magnetic resonance imaging (MRI); (d) normal pituitary–adrenal/pituitary–thyroid/pituitary–IGF-1 axis function; and (e) anosmia or hyposmia (6). An age-matched control group (healthy volunteers) with normal semen parameters was recruited from PUMCH. Semen samples were produced by masturbation after ≥3 days of abstinence.

### Materials and Instrument

The SPLASH LIPIDOMIX Mass Spec Standard was from AVANTI. Chromatographic grade methanol and methyl tert-butyl ether (MTBE) were purchased from the CNW Technologies, Shanghai, China. The Lipidyzer TM was from the AB Sciex Pte. Ltd., Massachusetts, United States. Chromatographic grade ammonium acetate, dichloromethane and isopropanol were obtained from the Merck Company, Darmstadt, Germany. The SCIEX ExionLC system was coupled to a SCIEX QTrap 6500+ (AB Sciex Pte. Ltd., Massachusetts, USA). ACQUITY UPLC HSS T3 (2.1 ∗ 100 mm, 1.8 μm, Waters Corporation, Milford, MA, USA) was used for lipidomics analysis.

### Sample Collection

The preparation before sampling, blood sample collection and specimen processing were conducted according to IFCC/C-RIDL protocols. Fasting blood samples were taken *via* venipuncture into Vacuette tubes containing procoagulant, and within 15–30 min after sample collection, the samples were centrifuged at 1,200×*g* for 10 min.

A semen sample from each participant was obtained by means of masturbation and ejaculation directly into noncytotoxic sterile containers. Freshly collected semen was liquefied for 30–60 min at room temperature and processed within 1 h of ejaculation for analysis of the sperm characteristics according to the criteria published by the WHO. The samples were centrifuged at 1,200×*g* for 10 min and frozen at −80°C to obtain 200 μl of seminal plasma.

### Laboratory Assays

All sex hormones were measured using an automated chemiluminescence immunoassay analyzer (Beckman Coulter UniCel DXI 800, Beckman Coulter; Brea, CA, USA) using the corresponding reagents, calibration materials and quality control materials. Sperm motility and concentration assessments were performed using computer-assisted sperm analysis systems (Suiplus SSA-II, Suiplus Software Co. Ltd.; Beijing, China). The microscope used with SSA-II was a Nikon 80i with a 20-phase objective.

### Sample Preparation for Metabolomics Profiling

Ten microliters of the seminal plasma was mixed with 190 μl of water in a sterile 1.5 ml Eppendorf tube, and 480 μl of extract solution (MTBE/MeOH = 5:1) with internal standard was added. The samples were vortexed for 60 s and sonicated for 10 min in an ice water bath. Then, the samples were centrifuged at 3,000 rpm for 15 min at 4°C, and 250 μl of supernatant was transferred to a fresh tube. Another 250 μl of MTBE was added to the rest of the sample, and the extraction step was repeated twice. The combined supernatants were freeze-dried and resuspended in 100 μl of DCM/MeOH/H_2_O (60/30/4.5) by sonication on ice for 10 min. The samples were centrifuged at 12,000 rpm for 15 min at 4°C, and 30 μl of the supernatant was transferred to a fresh glass vial for liquid chromatography-mass spectrometry (LC/MS) analysis.

### Quality Control

The quality control (QC) sample was prepared by mixing 15 μl of the supernatants from all of the seminal plasma samples. The QC sample was stored at −80°C and used according to the sequence.

### LC-MS/MS Analysis

A SCIEX ExionLC series UHPLC System was used to perform ultrahigh-performance liquid chromatography (UHPLC) separation. Mobile phase A consisted of water/acetonitrile (40%/60%) with 10 mmol/L ammonium acetate. Mobile phase B consisted of acetonitrile/isopropanol (10%/90%) with 10 mmol/L ammonium acetate. A gradient elution procedure was used (0.0–16.0 min, 80–2% mobile phase A; 16.01–18.0 min, 80% mobile phase A). The autosampler temperature was set as 6°C, and the injection volume was set as 2 μl. The column temperature was 40°C. The flow rate was set as 0.3 ml/min.

Assay development was performed with an AB Sciex QTrap 6500+ mass spectrometer. The ion spray voltage was +5,500/−4,500 V, ion source gas 1 was 50 psi, ion source gas 2 was 50 psi, the DP was ±80 V, the curtain gas was 40 psi, and the temperature was 350°C.

### Data Processing and Multivariate Data Analysis

Quantification of the target compounds was performed with Skyline 20.1 software. The absolute content of the individual lipids was calculated based on the peak area corresponding to the internal standard (IS) of the identical lipid class.

### Statistical Analysis

Experimental values are expressed as the mean ± SD. SIMCA software was used to perform statistical analysis. Variable importance in the projection (VIP) >1 and p <0.05 (Student’s t test) were used to screen the significance of metabolite levels. A Euclidean distance matrix was calculated to cluster the differential lipids with a complete linkage method.

## Results

### Baseline Clinical Characteristics of Patients With KS

All of the patients were ethnically Han Chinese (100%). Their average age was 22.2 ± 5.1 years old. All of the patients showed anosmia (21/21), and many patients had semen non-liquefaction (13/21) and condensed semen (12/21). Patients with KS showed lower sperm counts (13.9 ± 21.5 ∗ 10^6^), sperm concentrations (5.7 ± 9.8 ∗ 10^6^/ml) and sperm motility (7.4 ± 9.8%). The serum follicle-stimulating hormone (FSH), luteinizing hormone (LH) and testosterone (T) levels were significantly lower in the patients with KS than those in the control group (**Table 1**).

### Seminal Plasma Lipidomics

**Figure 1:**
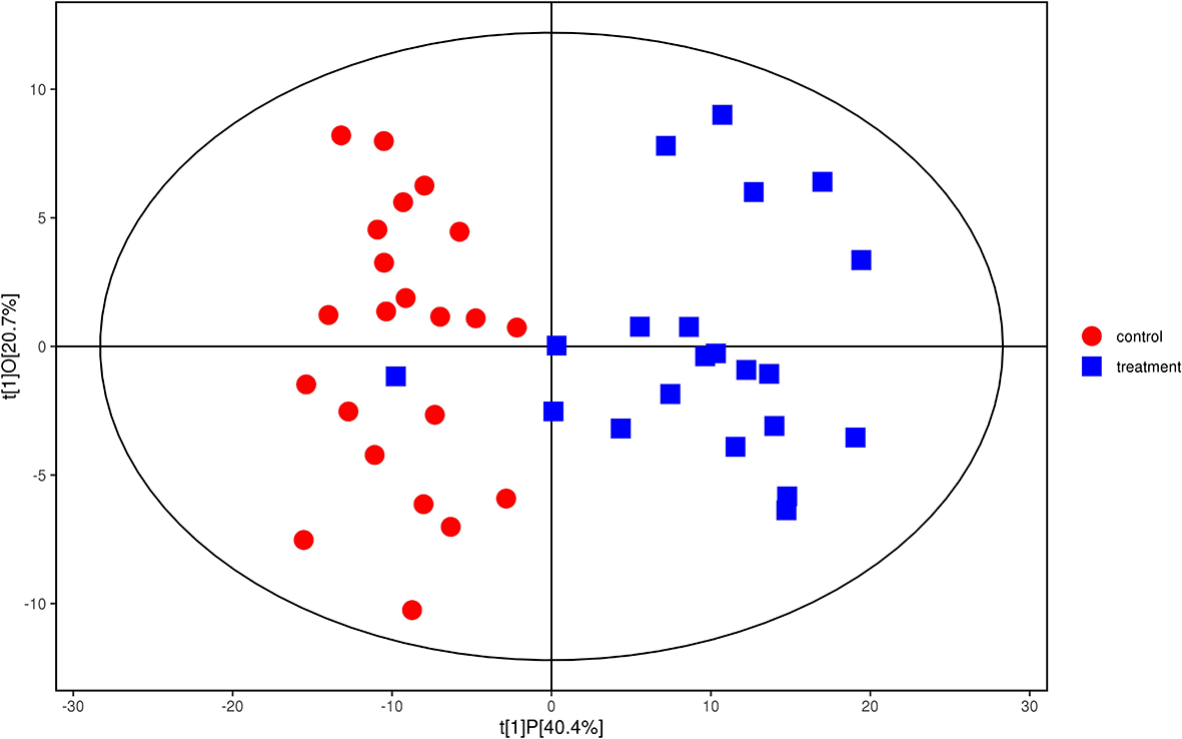
Orthogonal partial least squares-discriminant analysis (OPLS-DA) model for group treatment (KS, blue) *vs* control (HC, red). Ions marked in blue show a significant difference in intensity between the KS and HC groups.

**Figure 2:**
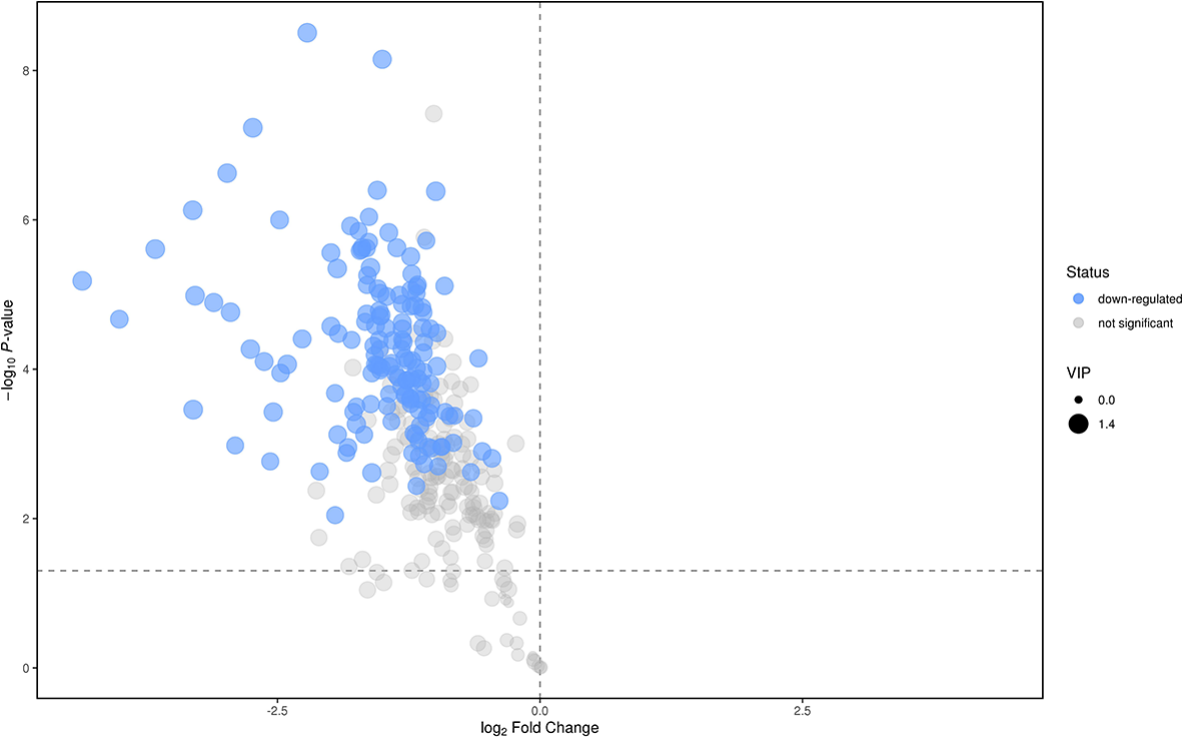
Volcano plot for the treatment (KS) *vs* control (HC) groups.

We analyzed the lipidomics data to discover lipid species related to KS. The effects of high variance of the variables and noise were minimized using scaled and logarithmic transformations. SIMCA software (V16.0.2, Sartorius Stedim Data Analytics AB, Umea, Sweden) was used to generate orthogonal projections to latent structures-discriminant analysis (OPLS-DA) plots (**Figure 1**). The OPLS-DA chart shows that the separation of the control group (HC, red) and the treatment group (patients with KS, blue) had a small overlap. Furthermore, a volcano plot was used to screen the statistically significant lipids (**Figure 2**), and blue dots indicate the presence of significantly downregulated metabolites in patients with KS. The value of the first principal component from the OPLS-DA analysis was obtained. The criterion of significantly changed metabolites was set as variable importance in the projection (VIP) >1 and p <0.05 (Student’s t test).

**Figure 3:**
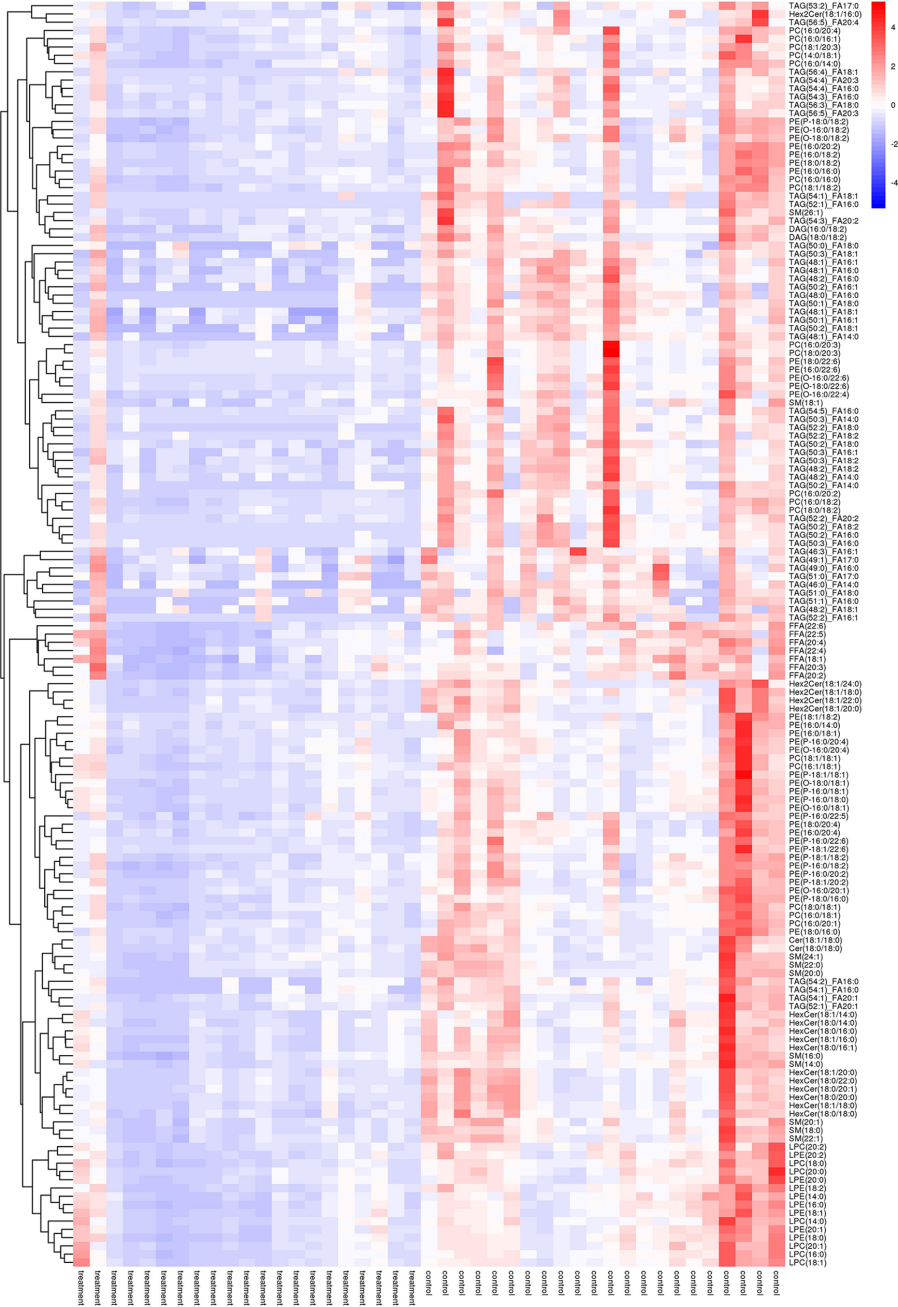
Heatmap of hierarchical clustering analysis for the treatment (KS) *vs* control (HC) groups.

**Figure 4:**
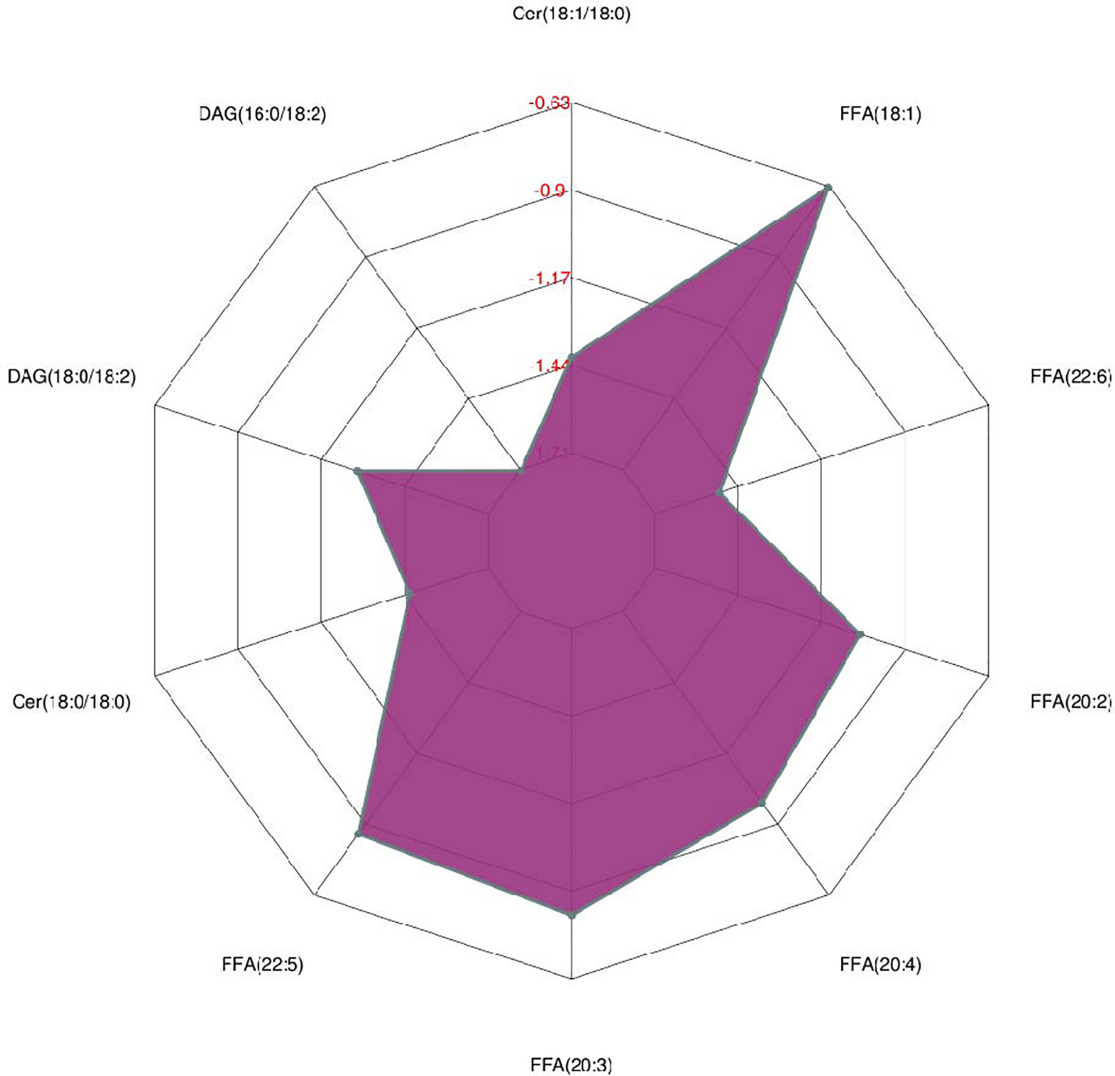
Radar chart analysis for the treatment (KS) *vs* control (HC) groups.

**Figure 5:**
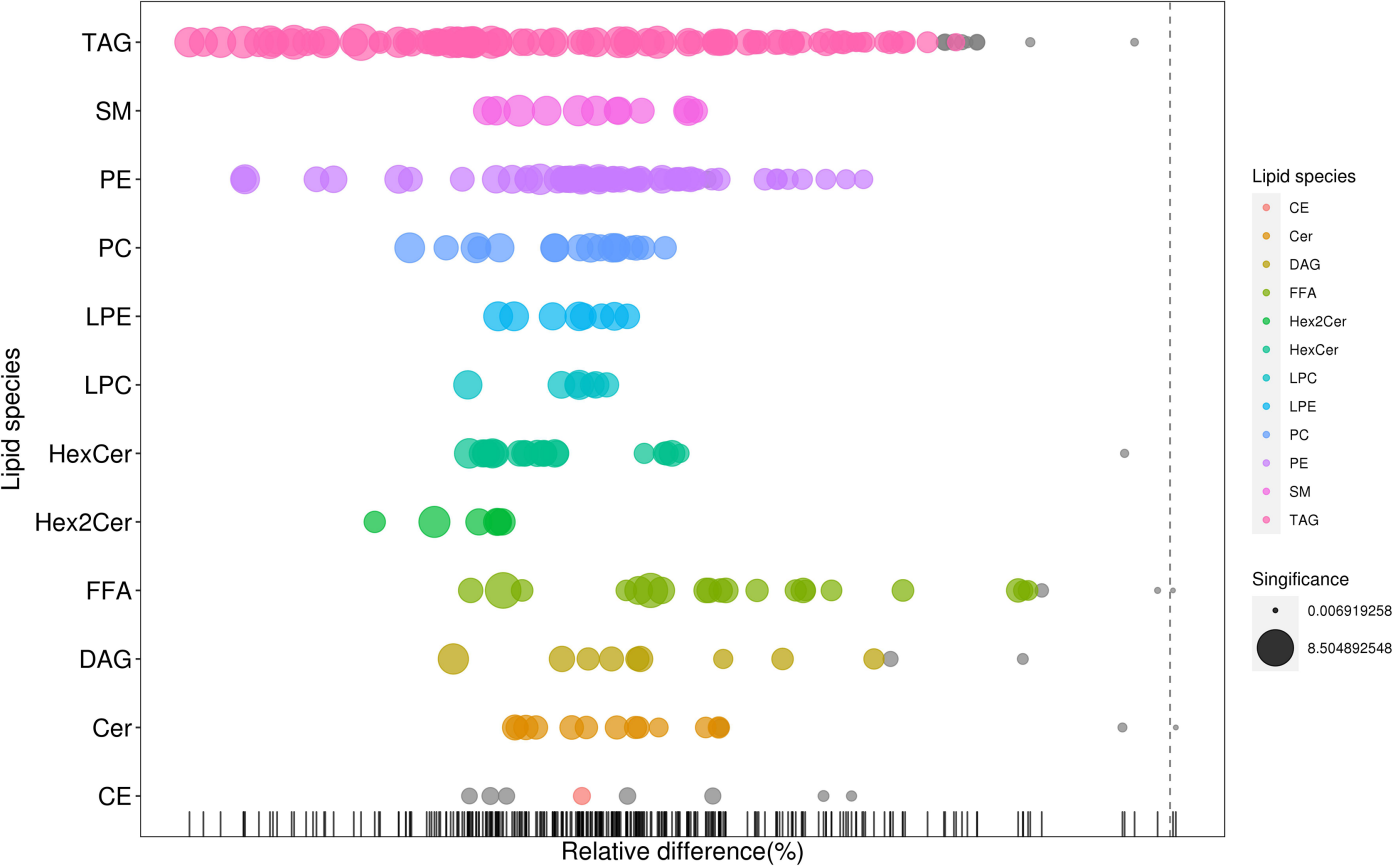
Bubble plot for the treatment (KS) *vs* control (HC) groups.

As shown in **Figure 2**, many lipid species such as triacylglycerol (TAG), sphingomyelin (SM), phosphatidylethanolamine (PE), lyso phosphatidylethanolamine (LPE), free fatty acid (FFA) and hexosylceramides (HexCer) changed in the patients with KS, TAG(53:2)_FA 17:0, TAG(56:5)_FA20:4, TAG(56:4)_FA18:1, TAG(54:4)_FA20:3, TAG(54:4)_FA16:0, PC (16:0/20:4), PC (16:0/16:1), PC (18:1/20:3), PC (14:0/18:1), and PC (16:0/14:0) decreased in patients with KS. Then, we calculated the Euclidean distance matrix, clustered the differential lipids with a complete linkage method, and displayed them in a heat map (**Figure 3**). The Euclidean distance matrix can be regarded as a weighted form of the adjacency matrix, which includes two levels of information: (1) whether the elements (points) are connected to each other and (2) the cost or distance of the connectivity between the elements (points). Therefore, the Euclidean distance matrix can be used to perform hierarchical cluster analysis. The ratio of the quantitative value of the different lipids was calculated using logarithmic conversion, and the corresponding content trend changes are displayed in the radar chart (**Figure 4**). For each group of comparisons, we calculated the corresponding ratio of the quantitative value of the different metabolites and took the logarithmic conversion with base 2 as the logarithmic conversion. The figure is represented with red font, each grid line represents a difference multiple, and the purple shading is represented by the connection of the lines of the difference multiples of each substance. The corresponding content trend changes are displayed in the radar chart. As shown in **Figure 4**, free fatty acids (FFAs) and diacylglycerols (DAGs) were downregulated in patients with KS. We also used a lipid group bubble plot to display the metabolite content change degree, difference significance and classification information (**Figure 5**). Each point in the lipid group bubble chart represents a metabolite. The size of the point represents the P-value of Student’s t test. The larger the point, the smaller the P-value. Gray dots represent nonsignificant differences with a P-value of not less than 0.05, and colored dots represent significant differences with a P-value of less than 0.05 (different colors are marked according to lipid classification). The abscissa of the lipid group bubble chart represents the percent relative change of the content of each substance in the group. The ordinate of the lipid group bubble chart represents the lipid classification information. The black line at the bottom shows the distribution density of the metabolites (one line represents one metabolite). As shown in **Figure 5**, TAGs, PCs and PE were the main differential species.

## Discussion

KS is a rare disease lacking specific markers for early diagnosis. In our previous work, we used an untargeted metabolomics approach and found that linoleic acid metabolism and glycerophospholipid metabolism changed substantially in KS (12). In this project, we used lipidomics analysis for further study. Many lipid species, such as triacylglycerols (TAGs), sphingomyelin (SM), phosphatidylethanolamine (PE), lyso-phosphatidylethanolamine (LPE), free fatty acids (FFAs) and hexosylceramides (HexCers), changed in patients with KS, and TAGs, PCs and PE were the main differential species.

Triacylglycerols (TAGs) are important lipid components that have various fatty acyl groups with different chain lengths, degrees of unsaturation and fatty acid isomers. In the cell, triacylglycerols can be hydrolyzed to form free fatty acids (FFAs) and 2-monoacylglycerol, and free fatty acids can be metabolized to biologically active compounds, oxidized to provide energy, or resynthesized into triacylglycerols for storage. A previous study on complete fatty acid profiling revealed potential candidate markers of semen quality (13). Our data showed that TAG(50:2)_FA18:2,TAG(52:1)_FA16:0, TAG(50:2)_FA16:0,TAG(52:2)_FA18:0, TAG(52:2)_FA18:2, TAG(48:0)_FA16:0, TAG(50:3)_FA16:0, and TAG(54:1)_FA18:1 decreased in patients with KS, which is in accordance with previous findings (**Table S1**) (13–15). In addition, the different FFAs also decreased in patients with KS (**Figure 4**). We think that a lower level of TAGs, to a certain extent, reveals a microenvironment without adequate nutrition. In the study, the patients with KS had lower sperm counts (13.9 ± 21.5 × 10^6^/ml *vs* HC of 39 × 10^6^/ml), sperm concentrations (5.7 ± 9.8 × 10^6^/ml *vs* HC of 15 × 10^6^/ml) and sperm motility (7.4 ± 9.8% *vs* HC of 38 to 42%), which may be associated with the microenvironment.

Phosphatidylcholines (PCs) are a class of glycerophospholipids that, along with other phospholipids, account for more than half of the lipids in most membranes. For example, lysophospholipids are PCs that affect lysophospholipid receptors as lipid signaling regulators (16, 17). In our work, PC (16:0/20:4), PC (16:0/16:1), PC (18:1/20:3), PC (14:0/18:1), and PC (16:0/14:0) decreased in patients with KS, indicating diagnostic value. The change in phosphatidylcholine composition and the saturation of their fatty acids may cause the deterioration of sperm membranes. This is consistent with a previous report that showed that glycerylphosphorylcholine (GPC) had potential diagnostic value for infertile men (18–22).

Phosphatidylethanolamine (PE) is a major component of membranes of many species. PE (16:0/22:6) and PE (O-16:0/22:6) were the significantly different metabolites (**Table S1**). For example, 50% of the total PE lipids are PE plasmalogens, which represent a major source of arachidonic acid and play an important role in the inflammatory response and also cause semen non-liquefaction (13/21) and semen condensation (12/21) (23, 24).

## Conclusions

In conclusion, we presented lipidomics profiling of semen plasma in patients with KS, and the main differential metabolic pathways focused on TAGs, PCs and PE. The results from this new report are consistent with our previous work, suggesting the promising value of these biomarkers. However, the exact mechanism remains to be further elucidated. In addition, since this is a single-center study with a relatively small sample size, the performance of these biomarkers needs to be further evaluated.

## Data Availability Statement

The data that support the findings of this study are available on request from the corresponding authors. The data are not publicly available due to privacy or ethical restrictions.

## Ethics Statement

The studies involving human participants were reviewed and approved by institutional committee that protects human subjects from PUMCH. Written informed consent to participate in this study was provided by the participants’ legal guardian/next of kin.

## Author Contributions

(I) Conception and design: XL and YG. (II) Administrative support: YL. (III) Provision of study materials or patients: YG. (IV) Collection and assembly of data: XL and YG. (V) Data analysis and interpretation: XL. All authors contributed to the article and approved the submitted version.

## Funding

This work was supported in part by the National Nature Science Foundation of China (Nos. 81671618, 81871302), the CAMS Initiative for Innovative Medicine (2017-I2M-3-001), the CAMS Initiative for Innovative Medicine (2017-I2M-B and R-01), and the Beijing Key Clinical Specialty for Laboratory Medicine - Excellent Project (No. ZK201000).

## Conflict of Interest

The authors declare that the research was conducted in the absence of any commercial or financial relationships that could be construed as a potential conflict of interest.

## Publisher’s Note

All claims expressed in this article are solely those of the authors and do not necessarily represent those of their affiliated organizations, or those of the publisher, the editors and the reviewers. Any product that may be evaluated in this article, or claim that may be made by its manufacturer, is not guaranteed or endorsed by the publisher.

## References

[1] Schwanzel-Fukuda M Bick D Pfaff DW. Luteinizing Hormone-Releasing Hormone (LHRH)-Expressing Cells Do Not Migrate Normally in an Inherited Hypogonadal (Kallmann) Syndrome. Brain Res Mol Brain Res (1989) 6:311–26. 10.1016/0169-328X(89)90076-4 2687610

[2] Pitteloud N Meysing A Quinton R Acierno JS Crowley WF. Mutations in Fibroblast Growth Factor Receptor 1 Cause Kallmann Syndrome With a Wide Spectrum of Reproductive Phenotypes. Mol Cell Endocrinol (2006) 254-255:60–9. 10.1016/j.mce.2006.04.021 16764984

[3] Maione L Dwyer A Francou B Guiochon-Mantel A Binart N Bouligand J. Genetic Counseling for Congenital Hypogonadotropic Hypogonadism and Kallmann Syndrome: New Challenges in the Era of Oligogenism and Nextgeneration Sequencing. Eur J Endocrinol (2018) 178:R55–80. 10.1530/EJE-17-0749 29330225

[4] Caronia LM Martin C Welt CK Sykiotis GP Quinton R Thambundit A. A Genetic Basis for Functional Hypothalamic Amenorrhea. N Engl J Med (2011) 64:215–25. 10.1056/NEJMoa0911064 PMC3045842 21247312

[5] Kaplan JD Bernstein JA Kwan A Hudgins L. Clues to An Early Diagnosis of Kallmann Syndrome. Am J Med Genet A (2010) 152A:2796–801. 10.1002/ajmg.a.33442 20949504

[6] Nie M Yu B Chen R Sun B Mao J Wang X. Novel Rare Variants in FGFR1 and Clinical Characteristics Analysis in a Series of Congenital Hypogonadotropic Hypogonadism Patients. Clin Endocrinol (Oxf) (2021) 00:1–10. 10.1111/cen.14436 33548149

[7] Nicholson JK Lindon JC. Systems Biology: Metabonomics. Nature (2008) 455:1054–6. 10.1038/4551054a 18948945

[8] Wu H Xue R Tang Z Deng C Liu T Zeng H. Metabolomic Investigation of Gastric Cancer Tissue Using Gas Chromatography/Mass Spectrometry. Anal Bioanal Chem (2010) 396:1385–95. 10.1007/s00216-009-3317-4 20012946

[9] Falsi g AML Gleerup CS Knudsen UB. The Influence of Omega-3 Fatty Acids on Semen Quality Markers: A Systematic PRISMA Review. Andrology (2019) 7:6. 10.1111/andr.12649 31116515

[10] Zanetti SR Monclus MdLÁ Rensetti DE Fornés MW Aveldaño MI. Differential Involvement of Rat Sperm Choline Glycerophospholipids and Sphingomyelin in Capacitation and the Acrosomal Reaction. Biochimie (2010) 92(12):1886–94. 10.1016/j.biochi.2010.08.015 20850501

[11] Boguenet M Bocca C Bouet P-E Serri O Chupin S Tessier L. Metabolomic Signature of the Seminal Plasma in Men With Severe Oligoasthenospermia. Andrology (2020) 8:1859–66. 10.1111/andr.12882 32770844

[12] Guo Y Li X Yan S Li Y. Metabolomic Alterations Associated With Kallmann Syndrome. Ann Transl Med (2020) 8(8):529. 10.21037/atm.2020.04.03 32411752 PMC7214890

[13] Zerbinati C Caponecchia L Rago R Leoncini E Bottaccioli AG Ciacciarelli M. Fatty Acids Profiling Reveals Potential Candidate Markers of Semen Quality. Andrology (2016) 4(6):1094–101. 10.1111/andr.12236 27673576

[14] Mu H Porsgaard T. The Metabolism of Structured Triacylglycerols. Prog Lipid Res (2005) 44:430–48. 10.1016/j.plipres.2005.09.002 16269186

[15] Masaki H Kim N Nakamura H Kumasawa K Kamata E Hirano KI. Long-Chain Fatty Acid Triglyceride (TG) Metabolism Disorder Impairs Male Fertility: A Study Using Adipose Triglyceride Lipase Deficient Mice. Mol Hum Reprod (2017) 23(7):452–60. 10.1093/molehr/gax031 28510703

[16] Pietiläinen KH Róg T Seppänen-Laakso T Virtue S Gopalacharyulu P Tang J. Association of Lipidome Remodeling in the Adipocyte Membrane With Acquired Obesity in Humans. PloS Biol (2011) 9:e1000623. 10.1371/journal.pbio.1000623 21666801 PMC3110175

[17] Wymann MP Schneiter R. Lipid Signalling in Disease. Nat Rev Mol Cell Biol (2008) 9:162–76. 10.1038/nrm2335 18216772

[18] Vorkas PA Isaac G Holmgren A Want EJ Shockcor JP Holmes E. Perturbations in Fatty Acid Metabolism and Apoptosis Are Manifested in Calcific Coronary Artery Disease: An Exploratory Lipidomic Study. Int J Cardiol (2015) 197:192–9. 10.1016/j.ijcard.2015.06.048 26142205

[19] Vouk K Ribič-Pucelj M Adamski J Rižner TL. Altered Levels of Acylcarnitines, Phosphatidylcholines, and Sphingomyelins in Peritoneal Fluid From Ovarian Endometriosis Patients. J Steroid Biochem Mol Biol (2016) 159:60–9. 10.1016/j.jsbmb.2016.02.023 26921767

[20] Zhao YY Wang HL Cheng XL Wei F Bai X Lin RC. Metabolomics Analysis Reveals the Association Between Lipid Abnormalities and Oxidative Stress, Inflammation, Fibrosis, and Nrf2 Dysfunction in Aristolochic Acidinduced Nephropathy. Sci Rep (2015) 5:12936. 10.1038/srep12936 26251179 PMC4528220

[21] Ried JS Baurecht H Stuckler F Krumsiek J Gieger C Heinrich J. Integrative Genetic and Metabolite Profiling Analysis Suggests Altered Phosphatidylcholine Metabolism in Asthma. Allergy (2013) 68:629–36. 10.1111/all.12110 23452035

[22] Deepinder F Chowdary HT Agarwal A. Role of Metabolomic Analysis of Biomarkers in the Management of Male Infertility. Expert Rev Mol Diagn (2007) 7:351–8. 10.1586/14737159.7.4.351 17620044

[23] Bogatcheva NV Sergeeva MG Dudek SM Verin AD. Arachidonic Acid Cascade in Endothelial Pathobiology. Microvasc Res (2005) 69:107–27. 10.1016/j.mvr.2005.01.007 15896353

[24] Monk JM Turk HF Fan YY Callaway E Weeks B Yang P. Antagonizing Arachidonic Acid-Derived Eicosanoids Reduces Inflammatory Th17 and Th1 Cell Mediated Inflammation and Colitis Severity. Mediators Inflamm (2014) 2014:917149. 10.1155/2014/917149 25136149 PMC4127240

