# Supplementary figures and images for: Seminal Plasma Lipidomics Profiling to Identify Signatures of Kallmann Syndrome
Source: Front Endocrinol (Lausanne). 2021 Jul 29;12:692690. doi: 10.3389/fendo.2021.692690 (PMC8358976; doi:10.3389/fendo.2021.692690)

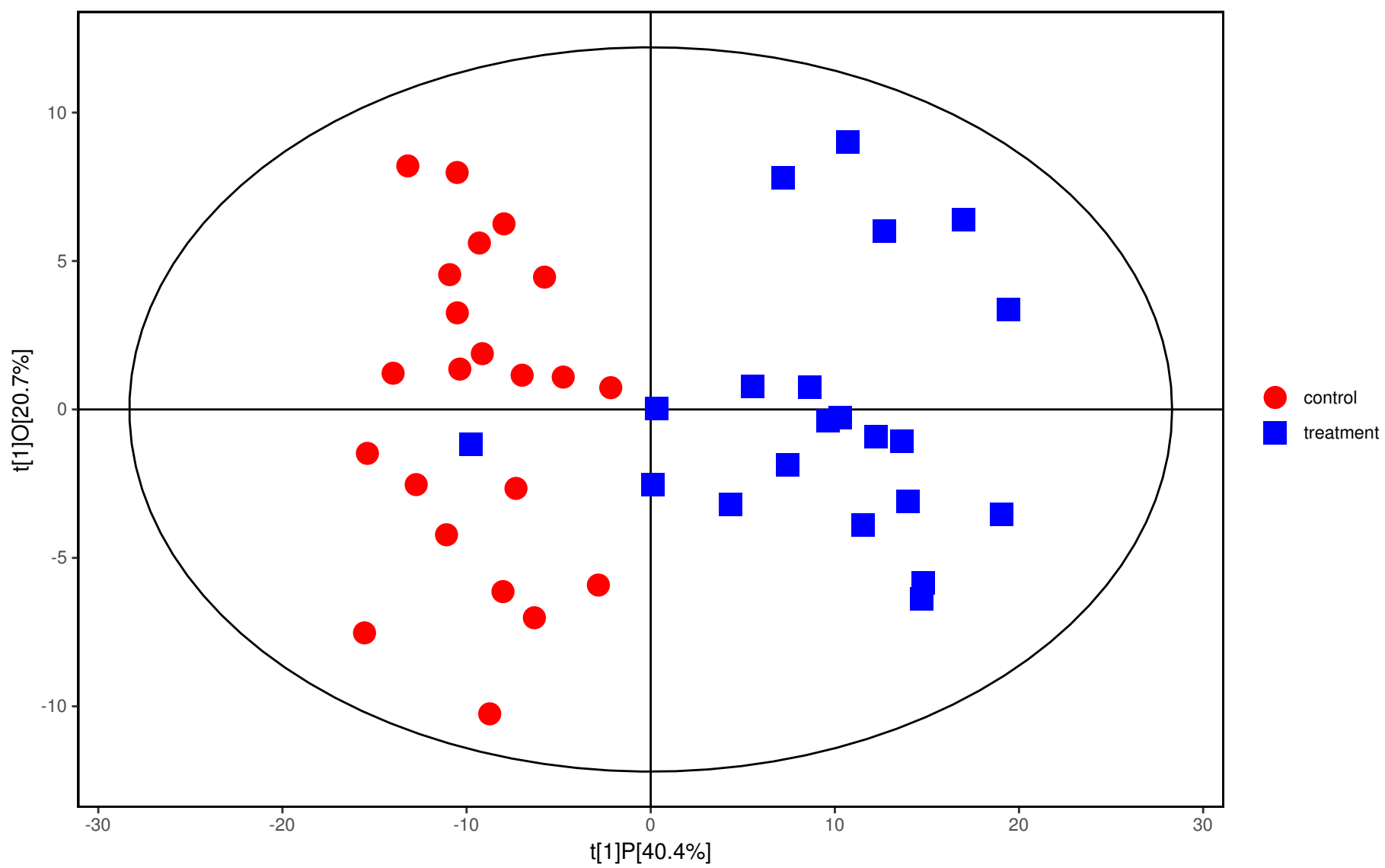

Supplement: Supplementary file 1 [file Image_1.pdf]
