# Supplementary material for: Seminal Plasma Lipidomics Profiling to Identify Signatures of Kallmann Syndrome
Source: Front Endocrinol (Lausanne). 2021 Jul 29;12:692690. doi: 10.3389/fendo.2021.692690 (PMC8358976; doi:10.3389/fendo.2021.692690)
Supplement: Supplementary file 2 [file Table_1.docx]

| **Name** | **Type** | **MEAN treatment** | **MEAN control** | **P-VALUE** | **Q-VALUE** | **FOLD CHANGE** | **LOG_FOLDCHANGE** |
| --- | --- | --- | --- | --- | --- | --- | --- |
| TAG(50:2)_FA18:2 | TAG | 18.28650284 | 375.2696242 | 6.5676E-06 | 3.12827E-06 | 0.048728972 | -4.359076416 |
| TAG(52:1)_FA16:0 | TAG | 26.69730745 | 428.9261374 | 2.14071E-05 | 6.07424E-06 | 0.062242202 | -4.00596308 |
| TAG(50:2)_FA16:0 | TAG | 73.0138456 | 925.2326613 | 2.46534E-06 | 1.74022E-06 | 0.078914038 | -3.663574222 |
| TAG(52:2)_FA18:0 | TAG | 20.82045562 | 206.0061474 | 7.44839E-07 | 1.1996E-06 | 0.101067157 | -3.306613844 |
| PE(16:0/22:6) | PE | 7.378817939 | 72.78345054 | 0.000348377 | 3.77448E-05 | 0.101380436 | -3.302148823 |
| PE(O-16:0/22:6) | PE | 26.70293997 | 260.1585282 | 1.03713E-05 | 3.92635E-06 | 0.102641033 | -3.284320505 |
| TAG(52:2)_FA18:2 | TAG | 14.8265203 | 127.7150099 | 1.28088E-05 | 4.48589E-06 | 0.116090664 | -3.10667614 |
| TAG(48:0)_FA16:0 | TAG | 211.3709203 | 1666.51177 | 2.37184E-07 | 6.44039E-07 | 0.12683434 | -2.978982692 |
| TAG(50:3)_FA16:0 | TAG | 20.25232483 | 156.1514351 | 1.7261E-05 | 5.36443E-06 | 0.129696694 | -2.946786394 |
| TAG(54:1)_FA18:1 | TAG | 8.916564594 | 66.67635801 | 0.001051073 | 8.81657E-05 | 0.133729029 | -2.902615428 |

Tabel S1 Top ten metabolites with significantly different abundance between group treatment (KS) and control (HC)
